# Cerebrovascular reactivity measurements using 3T BOLD MRI and a fixed inhaled CO_2_ gas challenge: Repeatability and impact of processing strategy

**DOI:** 10.3389/fphys.2023.1070233

**Published:** 2023-02-06

**Authors:** Emilie Sleight, Michael S. Stringer, Isla Mitchell, Madeleine Murphy, Ian Marshall, Joanna M. Wardlaw, Michael J. Thrippleton

**Affiliations:** ^1^ Centre for Clinical Brain Sciences, University of Edinburgh, Edinburgh, United Kingdom; ^2^ UK Dementia Research Institute, University of Edinburgh, Edinburgh, United Kingdom; ^3^ Edinburgh Imaging Facility, Royal Infirmary of Edinburgh, University of Edinburgh, Edinburgh, United Kingdom

**Keywords:** cerebrovascular reactivity, reliability, repeatability, blood oxygen-level dependent, hypercapnia

## Abstract

**Introduction:** Cerebrovascular reactivity (CVR) measurements using blood oxygen level dependent (BOLD) magnetic resonance imaging (MRI) are commonly used to assess the health of cerebral blood vessels, including in patients with cerebrovascular diseases; however, evidence and consensus regarding reliability and optimal processing are lacking. We aimed to assess the repeatability, accuracy and precision of voxel- and region-based CVR measurements at 3 T using a fixed inhaled (FI) CO_2_ stimulus in a healthy cohort.

**Methods:** We simulated the effect of noise, delay constraints and voxel- versus region-based analysis on CVR parameters. Results were verified in 15 healthy volunteers (28.1±5.5 years, female: 53%) with a test-retest MRI experiment consisting of two CVR scans. CVR magnitude and delay in grey matter (GM) and white matter were computed for both analyses assuming a linear relationship between the BOLD signal and time-shifted end-tidal CO_2_ (EtCO_2_) profile.

**Results:** Test-retest repeatability was high [mean (95% CI) inter-scan difference: −0.01 (−0.03, −0.00) %/mmHg for GM CVR magnitude; −0.3 (−1.2,0.6) s for GM CVR delay], but we detected a small systematic reduction in CVR magnitude at scan 2 versus scan 1, accompanied by a greater EtCO2 change [±1.0 (0.4,1.5) mmHg] and lower heart rate [−5.5 (−8.6,−2.4] bpm]. CVR magnitude estimates were higher for voxel- versus region-based analysis [difference in GM: ±0.02 (0.01,0.03) %/mmHg]. Findings were supported by simulation results, predicting a positive bias for voxel-based CVR estimates dependent on temporal contrast-to-noise ratio and delay fitting constraints and an underestimation for region-based CVR estimates.

**Discussion:** BOLD CVR measurements using FI stimulus have good within-day repeatability in healthy volunteers. However, measurements may be influenced by physiological effects and the analysis protocol. Voxel-based analyses should be undertaken with care due to potential for systematic bias; region-based analyses are more reliable in such cases.

## 1 Introduction

Cerebrovascular reactivity (CVR) reflects the ability of the cerebral blood vessels to dilate in response to a vasoactive stimulus. Investigating CVR impairment in cerebral tissues is of particular interest in patients with cerebrovascular diseases (Watchmaker et al., 2019; Blair et al., 2020). This can be achieved *in vivo* using magnetic resonance imaging (MRI) (Liu et al., 2019). Typically, a hypercapnic stimulus is used to trigger vasodilation and induce changes in vascular parameters, including cerebral blood flow (CBF), which can be detected using vascular-sensitive MRI techniques (Ogawa et al., 1990; Rostrup et al., 1994; Noth et al., 2008). The CVR MRI technique is well-correlated with other imaging modalities such as transcranial doppler and positron-emission tomography (Ziyeh et al., 2005; Heijtel et al., 2014). Blood oxygen-level dependent (BOLD) MRI is most widely used along with end-tidal carbon dioxide (EtCO_2_) recording, with CVR magnitude typically defined as the percent change in the BOLD signal due to the hypercapnic challenge divided by the change in EtCO_2_ to account for the magnitude of the stimulus (Sleight et al., 2021). As CVR reflects a dynamic process, CVR delay, which comprises CO_2_ travel time between the lungs and the brain tissues and vasodilation response time, is often included as a variable in the signal model to avoid underestimation of CVR magnitude (Liu et al., 2019). A haemodynamic response function (HRF) has been proposed as a means to separate travel to vasodilation response time (Poublanc et al., 2015; Yao et al., 2021), though this isn’t commonly used in the literature (Sleight et al., 2021).

Previous studies have found good repeatability of CVR magnitude measured with a hypercapnic BOLD experiment (Kassner et al., 2010; Leung et al., 2016; Dengel et al., 2017; Merola et al., 2018; Thrippleton et al., 2018; Hou et al., 2019), though it is lower in normal-appearing white matter (NAWM) than in grey matter (GM) (Kassner et al., 2010; Leung et al., 2016; Thrippleton et al., 2018). However, the studies did not report on inter-scan differences in physiological variables such as blood pressure, respiration and heart rates. Indeed, CVR is a momentary measurement affected by the underlying physiology: Changes in respiration rate can alter the arterial CO_2_ partial pressure (Petersson and Glenny, 2014) and studies have reported increased heart rate during hypercapnia (Grubb et al., 1974; Lipp et al., 2010). Repeatability of the CVR delay has only been reported for 1.5T MRI (Thrippleton et al., 2018), whereas CVR experiments are typically performed at 3T (Thrippleton et al., 2018). Furthermore, CVR data analysis methods differed across studies (Tong et al., 2011; Bright and Murphy, 2013; Bhogal et al., 2014; Donahue et al., 2016; Liu et al., 2017). First, various CVR delay computation methods were present across the literature: some studies used an estimated constant (Kassner et al., 2010; Leung et al., 2016; Dengel et al., 2017; Hou et al., 2019) and others a variable CVR delay (Thrippleton et al., 2018). It is unknown how robust the estimation of CVR delay is against noise and its impact on the reliability of CVR magnitude estimates. While voxel- (Kassner et al., 2010; Leung et al., 2016; Hou et al., 2019) and ROI-based (Dengel et al., 2017; Thrippleton et al., 2018) analyses were applied, there is little evidence to indicate which analysis is more appropriate.

In this work, we aimed to determine 1) the within-day test-retest repeatability of CVR magnitude and delay with a 3T BOLD MRI experiment using a fixed inhaled CO_2_ stimulus while measuring blood pressure, heart and respiration rates; 2) the impact of noise on accuracy and precision of CVR magnitude and delay measurements; 3) the impact of using variable versus fixed or zero delay; 4) the impact of extracting CVR magnitude and delay using voxel-based versus ROI-based analysis.

## 2 Materials and methods

### 2.1 Simulations

Simulations were conducted using MATLAB (version R2018b, MathWorks, Inc., MA, United States) to investigate the effect of noise and processing methods on CVR estimates. We simulated the EtCO_2_ trace as a block paradigm without noise ranging from 40 (normocapnia) to 50 mmHg (hypercapnia). Timings of the paradigm replicated the *in vivo* stimulus (see Section 2.4). The BOLD voxel time courses were generated by scaling and shifting the EtCO_2_ trace according to the given CVR magnitude and delay, respectively. BOLD signals had a temporal resolution of TR = 1 s. Values for the true median, mean and standard deviation of CVR magnitude and delay in ROIs were extracted from the healthy volunteer’s data acquired in this study by averaging CVR maps across subjects (see Supplementary Material).

The number of signals simulated for each ROI was set to the median number of voxels per ROI in a healthy volunteer dataset, described below, namely: 1,247 in subcortical GM (SGM), 656 in cortical GM (CGM) and 11,748 in NAWM. For each voxel, we added random Gaussian noise with a standard deviation equal to the hypercapnia-induced change in BOLD signal divided by the pre-defined temporal contrast-to-noise ratio (tCNR). The true CVR magnitudes and delays were sampled according to distributions of CVR magnitudes and delays extracted from the healthy volunteer data of this study (see Supplementary Material; Supplementary Figure S1). We therefore used distributions with a median/mean ± SD of 0.25/0.27 ± 0.13, 0.25/0.30 ± 0.21 and 0.10/0.11 ± 0.07%/mmHg for true CVR magnitude and 8/10 ± 8, 15/20 ± 16 and 27/29 ± 13 s for CVR delay in SGM, CGM and NAWM respectively.

For different values of tCNR and analysis type (voxel- and ROI-based), we simulated 1,000 repetitions (*N* voxels per repetition) of the same experiment. We computed the mean and standard deviation of CVR magnitude and delay estimates across the repetitions.

For the voxel-based analysis, we performed the multiple linear regression between each simulated BOLD time courses and time-shifted EtCO_2_ (see Section 2.5). For the ROI-based analysis, we applied the linear regression to the mean BOLD signal of the ROI.

To investigate the effect of the delay constraint, we repeated simulations using four delay ranges: −31–93, 0–58, −93–93, −31–124 s. We also simulated CVR magnitude estimation assuming a fixed delay, based on three previous approaches reported in the literature: 1) a global delay defined as the delay calculated from the averaged BOLD signal across GM and NAWM voxels, 2) a GM delay calculated from the averaged BOLD signal across GM voxels and 3) a delay of 0 s corresponding to no delay correction.

### 2.2 Participants

Healthy volunteers were recruited for two CVR scans. The study was conducted under Research Ethics Committee approval (ref. 14/HV/0001) and according to the principles expressed in the Declaration of Helsinki. All volunteers gave written informed consent. Exclusion criteria consisted of contraindication to MRI, migraine, hypertension, anxiety disorders, panic attacks, respiratory, and cardiovascular illnesses and known family history of subarachnoid haemorrhage, intracranial aneurysm or arteriovenous malformation.

### 2.3 Magnetic resonance imaging

All images were acquired using a 3T MRI scanner (MAGNETOM Prisma, Siemens Healthcare GmbH, Erlangen, Germany) with a 32-channel receive head coil. Each participant underwent two 13.5-min CVR scans, each acquired using axial 2D single-shot gradient-echo echo-planar imaging (GE-EPI; TR/TE = 1550/30 ms, 67°flip angle, 23.5 × 23.5 cm^2^ FOV, 94 × 94 acquisition matrix, 50 × 2.5 mm slices, 2.5 mm^3^ isotropic resolution, multiband acceleration factor 2, in-plane GRAPPA acceleration factor 2, 5 dummy scans) during a hypercapnic challenge. We discarded the first minute of the scanning (39 volumes) to obtain the same paradigm as in clinical studies on small vessel disease (Thrippleton et al., 2018). Structural imaging sequences consisted of sagittal T1-weighted 3D inversion recovery spoiled gradient-echo (T1W; TR/TE = 2500/4.37 ms, 7° flip angle, 25.6 × 25.6 × 19.2 cm^3^ FOV, 256 × 256×192 acquisition matrix size, 1.0 mm^3^ isotropic resolution, GRAPPA acceleration factor three in anterior-posterior phase-encoding direction) and axial T2-weighted 3D RARE (T2W; TR/TE = 3200/408 ms, 24.0 × 24.0 × 15.8 cm^3^ FOV, 256 × 256×176 acquisition matrix size, 0.9 mm^3^ isotropic resolution, GRAPPA acceleration factor 2 in phase- and partition-encoding directions).

The scanning session took place between 11 a.m. and 4 p.m. Structural images were acquired after the first CVR scan, followed by a short break when the subject came out of the scanner room before the second CVR scan.

### 2.4 Vasodilatory stimulus

The hypercapnic challenge used an established method which consisted of a block paradigm alternating between 2 min of medical air and 3 min of fixed inhaled CO_2_ stimulus for a duration of 12 min (Thrippleton et al., 2018). Actually, the first air block was 3 min long, but, for the analyses, we didn’t consider the data acquired during the first minute. The hypercapnic gas contained 6% CO_2_, 21% O_2_, and 73% N_2_, while the medical air contained 21% O_2_ and 79% N_2_ (BOC Special Products, United Kingdom). Expired CO_2_ and oxygen (O_2_) concentration waveforms were measured with a sampling frequency of 20 Hz using CD-3A CO_2_ and S-3A Oxygen sensors (AEI Technologies, Pittsburgh, United States) respectively, calibrated prior to each CVR scan. We recorded peripheral oxygen saturation, blood pressure pre- and post-CVR, heart rate and respiration rate using an MR conditional patient monitor with a sampling frequency of 1 Hz (MR400 and IP5; Philips, United Kingdom).

After each CVR scan, we asked participants to rate the tolerability of the scan (scale: 1 = very tolerable, 2 = tolerable, 3 = not very tolerable, 4 = intolerable) and recorded any reports of discomfort and possible hypercapnia symptoms.

### 2.5 Data processing

The CO_2_ and O_2_ concentrations were converted to partial pressures by multiplying them by the atmospheric pressure (i.e., 760 mmHg). The resulting waveforms were converted into EtCO_2_ and end-tidal oxygen (EtO_2_) traces using in-house MATLAB code, which identifies the signal peaks or troughs, as previously described (Thrippleton et al., 2018).

DICOM files were converted into NIFTI format (Li et al., 2016). BOLD images were realigned to each participant’s mean BOLD image using SPM12 (Friston, 2007).

**FIGURE 1:**
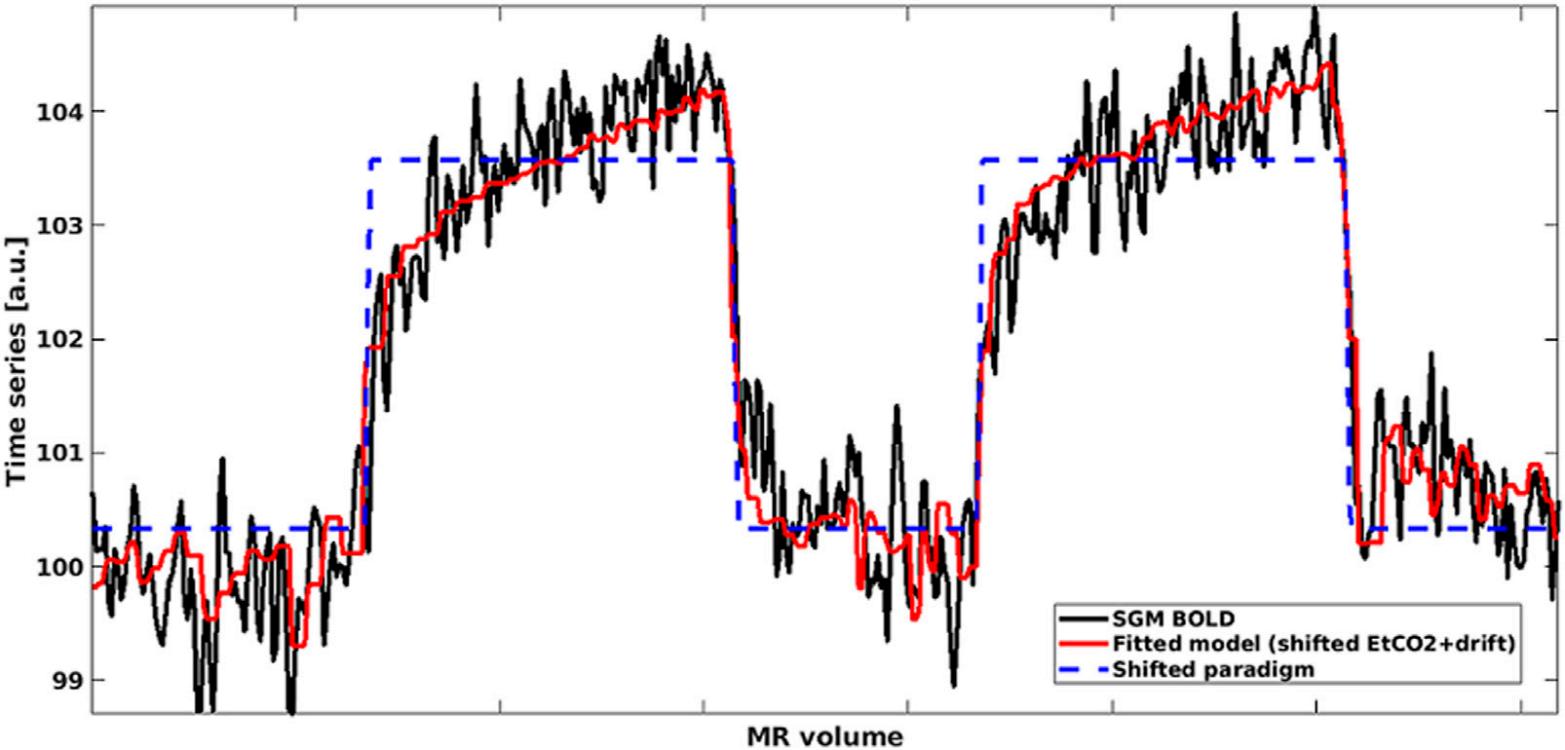
Mean BOLD time series in SGM (black) modelled using shifted EtCO_2_ and linear drift term (red). The shifted rescaled paradigm is shown in blue: High values correspond to hypercapnia periods whereas low values correspond to normocapnia periods. (SGM, Subcortical grey matter; EtCO_2_, End-tidal CO_2_).

For the ROI-based analysis, we computed the mean BOLD signal-time course in each ROI and performed multiple linear regression between the mean BOLD time course, the time-shifted EtCO_2_ course and a vector comprising the volume numbers to account for a linear signal drift in MATLAB (Figure 1). Unless otherwise mentioned, we allowed time shifts ranging from −31–93 s, both multiples of TR, for the EtCO_2_ profile allowing for long responses in damaged tissues (Thrippleton et al., 2018) and for negative delays in individual voxels or ROIs due to noise in the BOLD signals. This delay range was used previously (Thrippleton et al., 2018; Stringer et al., 2021). The optimal time shift was taken from the model with lowest sum of squared residuals. CVR magnitude (%/mmHg) was computed as the regression coefficient associated with the time-shifted EtCO_2_ term divided by the mean BOLD baseline signal and multiplied by 100, where the mean BOLD baseline was defined as the mean intensity of the mean BOLD signal over the first 30 volumes (overlapping medical air inhalation). CVR delay was defined as the optimal time shift plus 4 s to account for the sampling line delay. The latter was calculated prior to the study as the average time across five repetitions for an abrupt CO_2_ concentration change at the distal sampling point to be reported by the sensor. In the voxel-based analysis, the same multiple linear regression process was applied to the BOLD time course of each voxel within the ROI and the resulting CVR magnitude and delay estimates were averaged across the ROI.

For each analysis, we investigated the dependence of CVR estimates on the range of allowed delay values, i.e., delay constraint. In practice, delay constraint is limited by the amount of EtCO_2_ data recorded before and after the MRI acquisition. We reprocessed the data with a higher upper bound (from −31–124 s instead of −31–93 s).

We defined tCNR *in vivo* as the difference in mean BOLD intensity during hypercapnia and during normocapnia, excluding a 1 min transition period at the start of a new block to allow for the BOLD response to stabilise, divided by the standard deviation of the BOLD baseline signal. For each ROI, we computed the tCNR of the mean BOLD signal time course as well as the mean tCNR for voxels within the ROI.

### 2.6 Regions of interest

We used FSL FAST (Zhang et al., 2001) and FIRST (Patenaude et al., 2011) (FMRIB Analysis Group, Oxford, United Kingdom) to segment CGM, SGM (formed of the thalamus, putamen, pallidum and caudate nucleus) and NAWM in native T1W space based on the MNI-152 template (Fonov et al., 2009; Fonov et al., 2011). To reduce partial volume effects, all ROIs were eroded using a box kernel with a width of three voxels centred on target voxel. The linear affine transformation between the T1W and mean BOLD spaces was calculated using FLIRT (Jenkinson and Smith, 2001; Jenkinson et al., 2002) and applied to transform each ROI into the mean BOLD space. We then applied a threshold of 50% to obtain binary masks in the mean BOLD space.

### 2.7 Statistics

CVR magnitude, CVR delay and physiological parameters were reported as mean ± standard deviation. We investigated inter-scan, inter-block and inter-analysis differences using Bland-Altman statistics including the 95% confidence intervals for the mean difference reported in parentheses. We defined the inter-scan difference of a parameter as the parameter at scan 2 minus the parameter at scan 1. The inter-block difference in a parameter was defined as the mean value during the CO_2_ blocks minus the that during the air blocks. The inter-analysis difference of a parameter was defined as the value from the ROI-based analysis minus that from the voxel-based analysis. Furthermore, we computed the inter-scan coefficients of variation (CVs) for CVR magnitudes and delays as the standard deviation of the differences in paired measurements (i.e., one pair for each participant) divided by the mean of the pair-averaged values. The impact of physiological parameters was assessed using a linear regression model with the inter-scan difference in CVR magnitude as outcome, the inter-scan differences in baseline EtCO_2_ and EtCO_2_ change as independent variables and without an intercept, as described previously (Hou et al., 2019).

## 3 Results

### 3.1 Simulations

**FIGURE 2:**
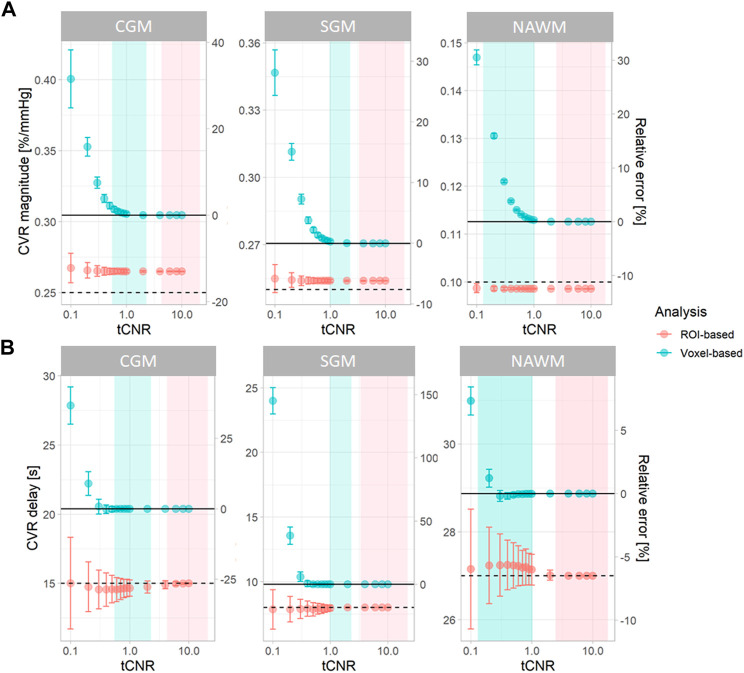
Simulations showing the effect of tCNR on the estimation of CVR magnitude **(A)** and delay **(B)** corresponding to all subcortical and cortical GM, and NAWM voxels. Data points and error bars indicate the mean ± the standard deviation of the estimates across 1000 simulations. Simulations were performed for both ROI- (pink) and voxel-based (blue) analyses. tCNR on the x-axis represents the tCNR in voxels. The ranges of tCNR values in the *in vivo* data are represented by the shaded areas in blue for tCNR in voxels and in pink for tCNR of mean BOLD signals. The delay constraint was from −31 to 93 seconds, corresponding to the delay constraint used to process real data. Horizontal solid and dashed lines represent the ground-truth mean and median values, respectively. The relative errors were computed with respect to the ground-truth mean values. (ROI: region of interest, CGM: cortical grey matter, GM: grey matter, NAWM: normal-appearing white matter, SGM: subcortical grey matter, tCNR: temporal contrast-to-noise ratio).

Simulations were performed using openly-accessible MATLAB scripts (https://doi.org/10.7488/ds/3503), which can also be used to process *in vivo* CVR data. At high tCNR, CVR parameters estimated from the simulated data using voxel-based analysis converged to the ground-truth mean values (Figure 2). However, at lower tCNR, random error increased and a positive bias was observed for both CVR magnitude and CVR delay for the voxel-based analysis. CVR magnitudes derived from ROI-based analysis had a similar precision to those from voxel-based analysis, but were always lower than the true CVR magnitude (Figure 2). CVR delays from ROI-based analysis converged to the ground-truth median values at high tCNR; at low tCNR, they remained accurate but were less precise.

**FIGURE 3:**
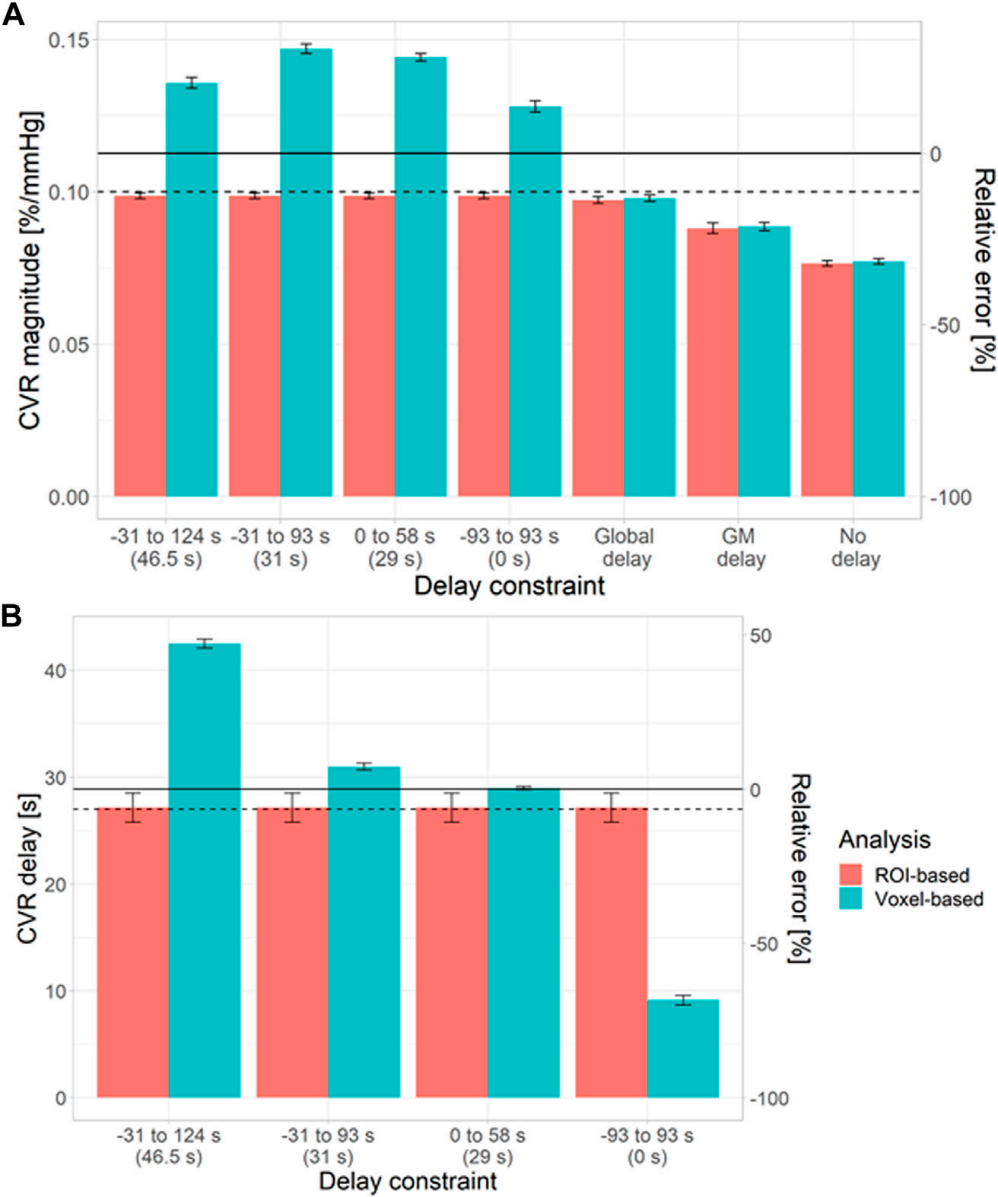
Simulations showing the impact of the delay constraint and assumption of fixed delay at a voxel tCNR of 0.1 on the estimation of CVR magnitude **(A)** and delay **(B)** in NAWM. The bars and error bars correspond to the mean and standard deviation respectively of the CVR estimates across 1000 repetitions for the voxel- (blue) and ROI-based (pink) analyses. Horizontal solid and dashed lines represent the ground-truth mean and median values, respectively. The relative errors were computed with respect to the ground-truth mean values. The central values for each delay constraint are shown in parentheses. (ROI: region of interest, GM: grey matter, NAWM: normal-appearing white matter).

To investigate the origins of the biases in the voxel-based analysis, we simulated NAWM CVR estimates using different delay constraints at a low tCNR of 0.1 (Figure 3). CVR estimates from ROI-based analysis were independent of the delay constraint. However, CVR estimates from voxel-based analysis depended on the centre of the delay constraint.

When CVR magnitude was estimated with a fixed delay, estimates from ROI- and voxel-based analyses were similar (Figure 3), but large systematic errors were observed (5–32%). The closer the fixed delay was to the mean delay in the ROI, the lower the relative error in CVR magnitude. For example, using a fixed delay obtained by fitting the mean GM signal resulted in large relative errors in NAWM CVR (>20%).

### 3.2 MRI experiments

Raw data acquired for this work were made openly accessible (https://doi.org/10.7488/ds/3492). We recruited 15 healthy volunteers (age: 28.1 ± 5.5 years, female: 53%) who all underwent the two CVR scans with a median time of 31.5 min between the starts of the scans. One participant didn’t complete the first CVR scan, but sufficient data was collected (4.2/12 min) to include in the analysis. Data from another subject were excluded from the analysis due to severe motion artefacts during scan one. Overall, the scans were well-tolerated (scan one: 4/15 very tolerable, 10/15 tolerable, 1/15 intolerable; scan two: 7/15 very tolerable, 8/15 tolerable). Symptoms reported by the subjects per scan were: 26/30 dyspnoea, 9/30 dry mouth, 8/30 dizziness or headache, 6/30 tingling sensations, 6/30 anxiety, 6/30 sensation of accelerated heart rate and 3/30 claustrophobia. Subjects reported mask discomfort in 3/30 scans and noticed a difference between the two inhaled gases in 27/30 scans.

### 3.3 CVR and physiological parameters

**FIGURE 4:**
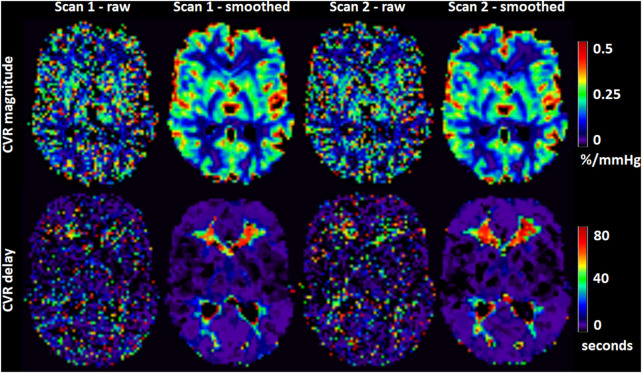
Maps of CVR magnitude and delay from one representative participant obtained from scan 1 and 2. The smoothed maps were obtained after spatially smoothing the BOLD volumes using a Gaussian filter with full width at half maximum of 4 mm and are shown here for visual purposes only.

**FIGURE 5:**
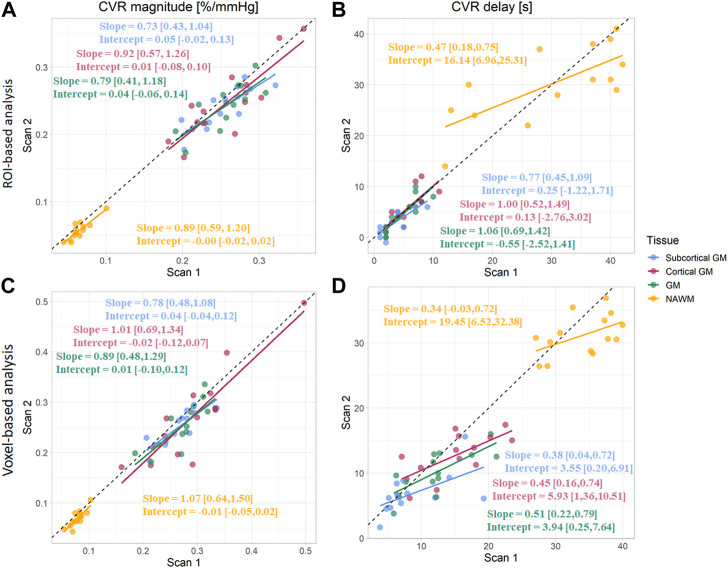
Comparison of CVR magnitudes **(A, C)** and delays **(B, D)** between scans. Estimates were computed in subcortical GM, cortical GM, GM, and NAWM from the ROI-based **(A, B)** and voxel-based analysis **(C, D)**. (GM, Grey matter; NAWM, Normal-appearing white matter).

**FIGURE 6:**
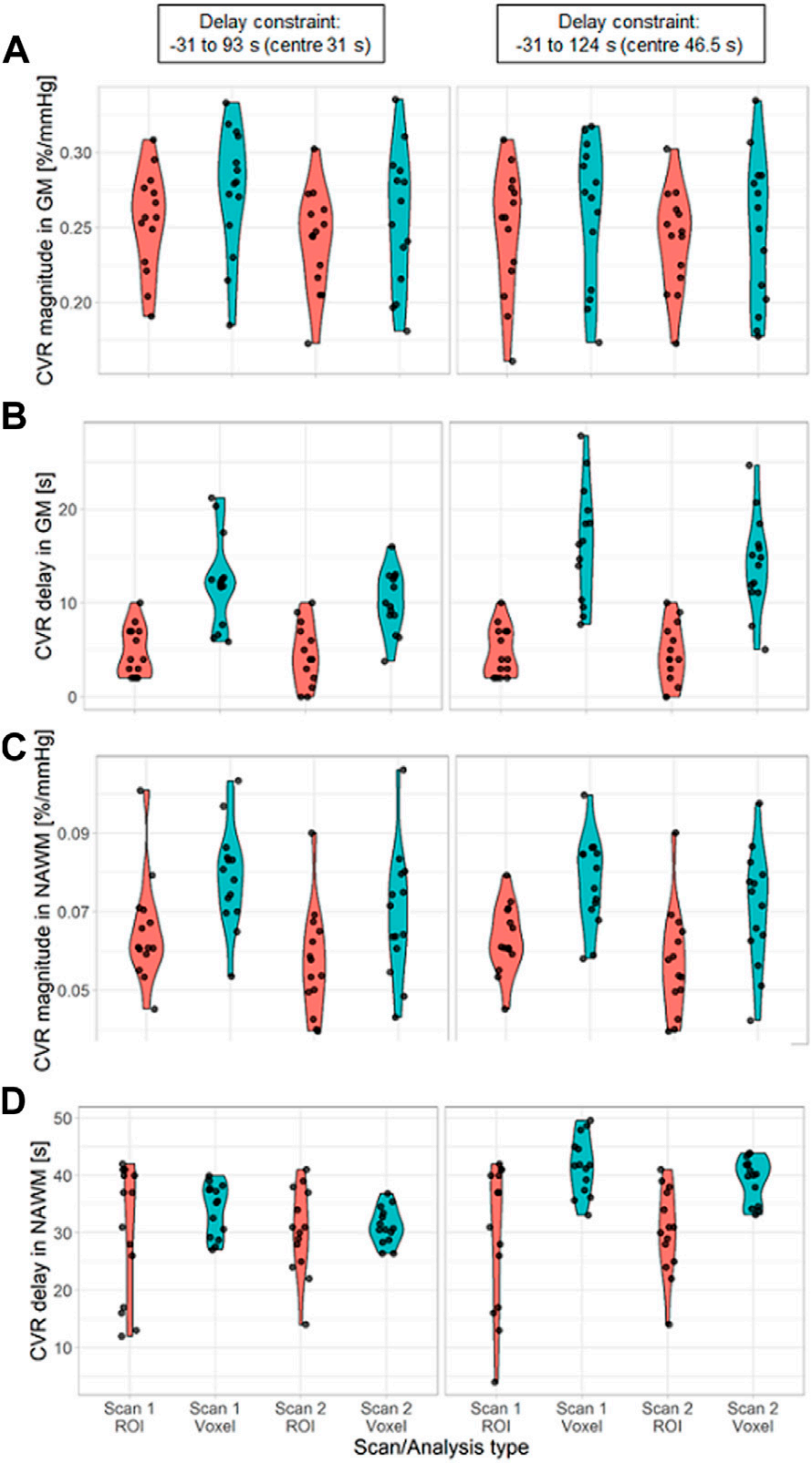
Violin distribution of CVR magnitude **(A, C)** and delay **(B, D)** in SGM **(A, B)** and NAWM **(C, D)** as a function of the scan and the processing method: ROI-based (red) versus voxel-based analysis (blue). The analysis was performed with two different delay constraints: −31–93 s (first column) and −31–124 s (second column). (SGM, Subcortical grey matter; NAWM, Normal-appearing white matter).

CVR magnitude and delay measurements at the two scans are shown in Table 1 and displayed in Figures 4–6; Supplementary Figure S2, S3. CVR magnitude was higher in GM than in NAWM [scan one difference: 0.19 (0.17, 0.21)%/mmHg for ROI-based analysis, 0.20 (0.17, 0.22)%/mmHg for voxel-based analysis], while CVR delay was shorter [−25.3 (−31.3, −19.3) s for ROI-based analysis, −21.8 (−23.7, −20.0) s for voxel-based analysis; Table 1]. Inter-scan CVs ranged from 7.48% to 14.91% for CVR magnitude and from 12.49% to 50.00% for CVR delay (Table 2). CVR magnitude was systematically lower at scan two than scan one [difference in GM: −0.01 (−0.03, −0.00)%/mmHg corresponding to −4% change for ROI-based analysis, −0.02 (−0.03, −0.00)%/mmHg corresponding to −7% for voxel-based analysis; Figures 5, 6; Supplementary Figure S2; Table 1].

**TABLE 1:** Mean and standard deviation across subjects of CVR magnitudes and delays in SGM, CGM, GM and NAWM computed for each scan with ROI- and voxel-based processing. Mean ± standard deviation of the inter-scan and inter-analysis differences are reported with the 95% confidence intervals in parentheses.

	ROI	Scan 1			Scan 2			Inter-scan difference (scan 2—scan 1)	
Analysis type		Voxel	ROI	ROI-voxel difference	Voxel	ROI	ROI-voxel difference	Voxel	ROI
CVR magnitude (%/mmHg)	SGM	0.26 ± 0.03	0.25 ± 0.04	−0.011 ± 0.004 (−0.014, −0.008)	0.25 ± 0.03	0.24 ± 0.03	−0.010 ± 0.003 (−0.012,-0.008)	−0.01 ± 0.02 (−0.03, −0.00)	−0.01 ± 0.02 (−0.02, −0.00)
	CGM	0.29 ± 0.08	0.26 ± 0.05	−0.030 ± 0.036 (−0.051, −0.010)	0.27 ± 0.09	0.25 ± 0.05	−0.022 ± 0.043 (−0.047, 0.003)	−0.02 ± 0.04 (−0.04, 0.01)	−0.01 ± 0.03 (−0.03, 0.01)
	GM	0.27 ± 0.04	0.25 ± 0.03	−0.020 ± 0.013 (−0.027, −0.013)	0.26 ± 0.05	0.24 ± 0.03	−0.014 ± 0.017 (−0.024, −0.004)	−0.02 ± 0.03 (−0.03, −0.00)	−0.01 ± 0.02 (−0.03, −0.00)
	NAWM	0.08 ± 0.01	0.07 ± 0.01	−0.014 ± 0.005 (−0.016, −0.011)	0.07 ± 0.02	0.06 ± 0.01	−0.012 ± 0.003 (−0.014, −0.010)	−0.010 ± 0.009 (−0.015, −0.004)	−0.008 ± 0.007 (−0.012, −0.004)
CVR delay (s)	SGM	8.8 ± 4.8	3.9 ± 2.6	−4.9 ± 4.3 (−7.4, −2.4)	6.9 ± 3.2	3.2 ± 2.4	−3.7 ± 2.1 (−4.9, −2.5)	−1.9 ± 3.9 (−4.1, 0.4)	−0.6 ± 1.4 (−1.5, 0.2)
	CGM	14.9 ± 5.3	5.4 ± 2.6	−9.6 ± 5.1 (−12.5, −6.6)	12.7 ± 3.4	5.5 ± 3.3	−7.2 ± 3.2 (−9.0, −5.3)	−2.3 ± 3.8 (−4.5, −0.1)	0.1 ± 2.0 (−1.0, 1.3)
	GM	12.2 ± 4.8	4.8 ± 2.7	−7.4 ± 4.3 (−9.9, −4.9)	10.1 ± 3.3	4.5 ± 3.2	−5.6 ± 1.9 (−6.8, −4.5)	−2.1 ± 3.2 (−3.9, −0.2)	−0.3 ± 1.5 (−1.2, 0.6)
	NAWM	34.0 ± 4.6	30.1 ± 11.4	−4.0 ± 10.1 (−9.8, 1.9)	31.2 ± 3.2	30.2 ± 7.4	−1.0 ± 7.2 (−5.1, 3.2)	−2.8 ± 4.1 (−5.2, −0.5)	0.1 ± 8.0 (−4.5, 4.7)

Abbreviations: CVR, cerebrovascular reactivity; GM, grey matter; SGM, subcortical GM; CGM, cortical GM; NAWM, normal-appearing white matter; ROI, region of interest.

**TABLE 2:** Inter-scan coefficients of variation for CVR magnitude and delay in SGM, CGM, GM, and NAWM as a function of the analysis type.

	Inter-scan CV for CVR magnitude [%]		Inter-scan CV for CVR delay [%]	
Analysis type	Voxel	ROI	Voxel	ROI
SGM	7.48	7.95	50.00	40.92
CGM	14.91	10.98	27.66	37.44
GM	10.34	8.82	28.87	33.18
NAWM	11.75	10.79	12.49	26.44

Abbreviations: CVR, cerebrovascular reactivity; CV, coefficient of variation; ROI, region of interest; GM, grey matter; SGM, subcortical GM; CGM, cortical GM; NAWM, normal-appearing white matter.

Physiological variables and baseline BOLD signals measured at both scans are given in Table 3. EtCO_2_ (Supplementary Figure S4; Table 3) and EtO_2_ (Table 3) changes were greater in scan two than in scan one [+1.0 (0.4, 1.5) mmHg for EtCO_2_, +1.6 (0.4, 2.8) mmHg for EtO_2_] but the baseline values were similar. In a linear model, reduced CVR magnitude was associated with increased EtCO_2_ change in SGM [−0.012 (−0.021, −0.003)%/mmHg^2^] and NAWM [−0.006 (−0.010, −0.002)%/mmHg^2^], and increased baseline EtCO_2_ in GM [−0.013 (−0.023, −0.004)%/mmHg^2^]. Mean heart rate was lower at scan two than at scan one [difference: −5.5 (−8.6, −2.4) bpm; Table 3]. Heart rate was lower during the CO_2_ block than during the air block in scan one but did not change between blocks in scan two. The respiration rate did not differ between scans and blocks nor were there differences in mean arterial pressure pre- and post-CVR scan (Table 3).

**TABLE 3:** Mean and standard deviation of the physiological variables. The mean and standard deviation of inter-scan and inter-block differences are reported, together with the 95% confidence intervals.

	ROI/Block	Scan 1	Scan 2	Inter-scan difference (scan 2—scan 1)
BOLD baseline (a.u)	SGM	340.1 ± 53.1	340.4 ± 50.7	0.4 ± 34.9 (−13.2, 13.9)
	CGM	328.9 ± 53.5	328.1 ± 49.2	−0.9 ± 35.3 (−14.6, 12.8)
	GM	336.4 ± 53.1	336.3 ± 49.3	−0.1 ± 35.3 (−13.8, 13.6)
	NAWM	295.4 ± 43.3	296.0 ± 39.9	0.6 ± 29.2 (−10.7, 11.9)
EtCO_2_ baseline (mmHg)	−	38.0 ± 3.8	37.7 ± 3.0	−0.3 ± 1.3 (−1.1, 0.4)
EtCO_2_ change (mmHg)	−	12.7 ± 1.6	13.6 ± 1.3	1.0 ± 1.0 (0.4, 1.5)
EtO_2_ baseline (mmHg)	−	122.8 ± 5.4	122.9 ± 4.2	0.1 ± 3.4 (−1.9, 2.1)
EtO_2_ change (mmHg)	−	14.0 ± 3.1	15.6 ± 2.4	1.6 ± 2.1 (0.4, 2.8)
MAP pre-CVR scan (mmHg)	−	84.7 ± 7.7	85.8 ± 9.4	1.14 ± 8.28 (−3.64, 5.93)
MAP post-CVR scan (mmHg)	−	78.6 ± 8.4	78.8 ± 9.2	0.14 ± 5.32 (−2.93, 3.21)
Difference in MAP (post-CVR—pre-CVR) (mmHg)	−	−6.02 ± 5.76	−7.02 ± 6.58	−1.00 ± 8.76 (−6.06, 4.06)
Heart rate (bpm)	Air	71.6 ± 13.0	65.5 ± 13.7	−6.1 ± 5.2 (−9.1, −3.1)
	CO_2_	70.1 ± 13.1	65.4 ± 13.6	−4.6 ± 5.8 (−8.0, −1.3)
	All	70.9 ± 13.0	65.5 ± 13.6	−5.5 ± 5.4 (−8.6, −2.4)
Respiration rate (breaths per minute)	Air	12.3 ± 3.6	12.4 ± 3.6	0.1 ± 1.0 (−0.5, 0.6)
	CO_2_	12.4 ± 3.5	12.5 ± 3.5	0.1 ± 1.3 (−0.6, 0.9)
	All	12.4 ± 3.5	12.4 ± 3.5	0.1 ± 1.0 (−0.5, 0.7)
Inter-block difference in heart rate (bpm) (CO_2_—Air)	−	−1.5 ± 2.3 (−2.8, −0.2)	−0.1 ± 1.9 (−1.2, 1.0)	−
Inter-block difference in respiration rate (breaths per minute) (CO_2_—Air)	−	0.1 ± 0.9 (−0.4, 0.6)	0.0 ± 0.9 (−0.5, 0.6)	−

Abbreviations: ROI, region of interest; BOLD, blood oxygen-level dependent; GM, grey matter; SGM, subcortical GM; CGM, cortical GM; NAWM, normal-appearing white matter; EtCO_2_, end-tidal carbon dioxide; EtO_2_, end-tidal oxygen; MAP, mean arterial pressure.

### 3.4 Comparison of ROI- and voxel-based analysis *in vivo*

CVR magnitude was lower and CVR delay shorter in the ROI-based versus voxel-based analysis [e.g., difference for scan 1: −0.020 (−0.027, −0.013)%/mmHg for GM CVR magnitude, −7.4 (−9.9, −4.9) s for GM CVR delay; Table 1]. Nevertheless, CVR magnitude had a good test-retest repeatability (CV: 7.48–14.91%) in all ROIs independent of analysis type (Figure 5; Table 1, 2). The repeatability of CVR delay in GM structures was good (28.87% for the voxel-based analysis, 33.18% for the ROI-based analysis; Figure 5 and Table 1, 2. CV for CVR delay in NAWM from the voxel-based analysis was 12.49%, compared to 26.44% in the ROI-based analysis (Table 2), and its distribution was tightly centred around 33 s (Figure 6).

Increasing the upper delay constraint resulted in a delay range with a higher mid-point (46.5 s for the −31–124 s range vs. 31 s for the −31–93 s range). CVR delays from the voxel-based analysis were extended [difference for scan 1: +4.1 (2.7, 5.4) s in GM] whereas CVR delays from the ROI-based analysis did not change (Figure 6; Supplementary Table S1). CVR magnitudes from the voxel-based analysis were lower [difference for scan one: −0.015 (−0.027, −0.003)%/mmHg in GM; Figure 6; Supplementary Table S1]; CVR magnitudes estimated using ROI analysis weren’t affected.

## 4 Discussion

In this work, we found through simulations that 3T BOLD CVR magnitude values determined using voxel-based analysis rapidly lost accuracy and precision at low tCNR. ROI-based analysis estimated CVR delay accurately but underestimated CVR magnitude due to averaging BOLD signals across voxels with a distribution of ground-truth values. Fitting data with a variable delay parameter was found to be essential to obtain accurate CVR magnitude estimates, however estimates from voxel-based analysis can be strongly dependent on the delay constraints. The 3T BOLD CVR experiment using a fixed inspired CO_2_ stimulus showed good within-day repeatability, though CVR delay estimates were less repeatable than CVR magnitude estimates. However, we noted small systematic differences in CVR magnitude between the two scans.

### 4.1 Simulations

Simulation results showed that, for ROI-based analysis, the accuracy of CVR magnitude estimates didn’t depend on tCNR, but there was a consistent underestimation with respect to the ground-truth mean (6%–26% depending on the ROI) due to signal averaging over voxels with a distribution of ground-truth CVR delay. On the other hand, CVR magnitude estimates were closer to the ground-truth median, although without converging towards it at high tCNR. Moreover, CVR delay estimates from ROI-based analysis were close to the ground-truth median value, independent of tCNR, reflecting the asymmetry of the CVR delay distribution. For voxel-based analysis, CVR magnitude and delay were accurate with respect to the ground-truth mean values at high tCNR. However, substantial bias with respect to the ground-truth mean CVR magnitude and delay values were found at low tCNR. Additional simulations showed that the choice of delay constraints had a strong impact on the accuracy of both parameters at low tCNR. This can be explained by the distribution of the CVR delay estimates being dominated by the delay constraints at low tCNR. Regarding precision, this was similar for CVR magnitude estimates using both methods. For CVR delay estimates, precision was higher for voxel-based analysis, however this may reflect the estimates being determined by the constraints rather than the intrinsic precision. Additional simulations explored the practice, reported in the literature, of fitting the CVR signal without a delay, or with a delay obtained by fitting the mean signal in GM or across the whole brain. This resulted in substantial systematic errors (2–24%) in CVR magnitude, irrespective of whether voxel- or ROI-based analysis was used, though precision was increased.

### 4.2 *In vivo* findings

In our group of healthy volunteers, we found GM CVR magnitudes (range: 0.19%—0.24%/mmHg) within the range of reported values from previous repeatability studies (Kassner et al., 2010; Leung et al., 2016; Thrippleton et al., 2018), though one study using an EtCO_2_ targeting stimulus reported GM CVR magnitude of 0.43%/mmHg (Dengel et al., 2017). In our study, NAWM CVR magnitudes were lower than in GM (0.06—0.08 versus 0.19%—0.24%/mmHg); these values were also in good agreement with the literature (Kassner et al., 2010; Thrippleton et al., 2018), except for one study that reported NAWM CVR magnitude of 0.15%/mmHg, but scanned adolescents, used an EtCO_2_ targeting stimulus and processed the data using a voxel-based analysis with fixed global delay (Leung et al., 2016). CVR delays were shorter in GM than in NAWM (3.2—14.9 s versus 30.1—34.0 s), agreeing with results from a previous repeatability study at 1.5T (Thrippleton et al., 2018). Our results also provide further evidence for the good repeatability of the CVR experiment using an FI stimulus: CVs for CVR magnitude repeatability were low (range including all ROIs: 7.48%—14.91%), similar to reported literature values using the same technique and processing method (Thrippleton et al., 2018), but higher than studies using an EtCO_2_ targeting stimulus (Kassner et al., 2010; Dengel et al., 2017). CVs for CVR delay repeatability (12.49%—50.00%) were similar to previously reported values at 1.5T (Thrippleton et al., 2018).

When comparing analysis approaches, we found systematically higher CVR magnitudes and longer CVR delays in all tissues for the voxel-based analysis, which was consistent with simulations. Indeed, simulations showed that processing methods are intrinsically dependent on the ground-truth distributions and differences in CVR estimates obtained from voxel- and ROI-based analyses can arise from asymmetry in those distributions. This finding, confirmed here in simulations and in data from healthy volunteers, can limit inter-study comparisons. Repeatability of CVR magnitude measurements was similar for both methods, however the repeatability for CVR delay in NAWM computed using a voxel-based analysis was higher than for ROI-based analysis. We showed that these effects were related to the delay constraint and high noise level in the voxel-wise BOLD signals. These findings are consistent with our simulation results and show that CVR estimates from voxel-based analysis are likely to be unreliable.

Surprisingly, average CVR magnitude was slightly lower at the second scan. Such a bias was only reported in one previous repeatability study, which found that a reduction in CVR magnitude between scans was associated with higher baseline EtCO_2_ and greater EtCO_2_ change in the second scan (Hou et al., 2019). In the present study, EtCO_2_ change was also significantly greater in the second scan compared to the first scan, but baseline EtCO_2_ was unchanged. Greater EtCO_2_ change was also associated with reduction in CVR magnitude. Therefore, we speculate that the non-linearity of the (approximately sigmoidal (Bhogal et al., 2014)) BOLD response to EtCO_2_ combined with the greater EtCO_2_ change explained the reduced CVR estimates based on a linear model at the second scan. The reason for the greater EtCO_2_ change at the second scan isn’t known; based on the inter-scan reduction in heart rate, we speculate that habituation to the CVR experiment could be a contributing factor. This could impact studies where CVR scans are repeated within the same day, for example to investigate instantaneous effects of a specific treatment on CVR. On the other hand, this might be a short-term effect that would not affect longitudinal studies of CVR, but further work is needed to confirm this.

### 4.3 Implications

Our findings have some implications for future studies. First, using devices targeting specific values of and changes in EtCO_2_ could be beneficial in reducing the impact of variable EtCO_2_ change and baseline EtCO_2_ (Fierstra et al., 2013). Familiarising the participants with the gas challenge prior to the CVR scan is essential to avoid anxiety and to minimise differences in baseline physiological state between repeated CVR measurements. Measuring blood pressure, heart and respiration rates during the CVR scan provides an indication of the extent of these effects.

The dynamic aspect of the CVR response should be taken into account. Estimating CVR delay provides additional physiological information, and is known to differ between tissues (Thomas et al., 2014; Thrippleton et al., 2018) and diseases (Thrippleton et al., 2018; Atwi et al., 2019). However, the interpretation of CVR delay should take into account possible steal effects through the redistribution of blood flow from blood vessels that have more reactivity (Sobczyk et al., 2014; Bhogal et al., 2015) and venous architecture in the WM that contributes to delayed WM responses (Bhogal, 2021). A recent systematic review showed that, though correcting for CVR delay is becoming more common, consensus regarding the delay extraction method is still missing (Sleight et al., 2021). We found that variable delays should be used when modelling the signal, since fitting with a fixed delay may result in large biases. Delay constraints are needed to restrict delays to realistic values. However, to allow variable delays, one should ensure sufficient EtCO_2_ recording before and after the CVR scan. We expect that the findings of this study would also be applicable in analyses where bulk alignment of the EtCO_2_ with the mean whole-brain or mean GM BOLD signal is used as a pre-processing step followed by voxel- or ROI-based analysis of CVR delay and magnitude.

Potential asymmetry in the distributions of ground-truth parameters should be considered. Using the median instead of mean estimates across ROIs in a voxel-based analysis could be more representative of the underlying distribution.

It is important to achieve sufficiently high tCNR to ensure accurate voxel-based mapping of CVR parameters. If this cannot be achieved then it may be advantageous to use a ROI-based analysis. However, intra-regional heterogeneity of the CVR parameters will be missed using this method, and the CVR magnitude estimates may not reflect the true mean values of non-uniform distribution. Moreover, any processing method that uses a voxel-based analysis to determine CVR delay will be subject to the bias at low tCNR found in this study (e.g., determining CVR delay for each voxel in order to realign the MRI signal before averaging). Analysis of data using different delay constraints may help verify that the estimates are not biased in this way. In our healthy volunteer cohort, analysis using different constraints did affect the results, implying that a ROI-based analysis could be more accurate in this context.

### 4.4 Strengths and limitations

A strength of this work is that we used simulations to compare different analysis types, tCNR levels and fitting constraints. As such, we could predict estimation errors based on known ground-truth values. Furthermore, this is the first study to objectively compare multiple analysis approaches that have been commonly reported in the literature. Lastly, our *in vivo* repeatability MRI dataset and simulation code have been made publicly accessible to allow other researchers to test and compare their processing methods; it is hoped that this will facilitate objective comparison of methods and development of consensus-based harmonisation in the field.

There are some limitations in this study. First, findings from this study might not be applicable to CVR MRI experiments where EtCO_2_ trace is not available. Furthermore, we didn’t investigate the effect of pre-processing methods such as spatial smoothing. However, we expect those to have an impact on the accuracy, precision and specificity of CVR estimates which should be investigated in future work. Regarding the simulations, we did not account for a non-instantaneous impulse response function (Poublanc et al., 2015). Research is on-going to determine and validate the HRF underlying the BOLD CVR signal (Poublanc et al., 2015; Yao et al., 2021). This could be addressed in future work. Moreover, the simulated noise was assumed to be independent in each voxel. For *in vivo* data, some noise components are temporally and spatially correlated due to phenomena such as physiological noise and patient motion. Future work could benefit from the development of a realistic four-dimensional digital reference object for CVR. We also assumed no correlation between ground-truth CVR magnitude and delay within each tissue, since the relationship isn’t known. Furthermore, while our simulations incorporated non-uniform distributions of ground-truth CVR magnitude and delay within tissues, these were based on average measurements from our relatively small healthy volunteer cohort, which are themselves subject to the limitations addressed in this work. Future simulation work could incorporate improved estimates of ground-truth CVR parameter distributions. This limitation may affect the quantitative results but is unlikely to affect our conclusions.

### 4.5 Conclusion

In conclusion, we have shown that CVR measurement using 3T BOLD MRI with a fixed inhaled CO_2_ concentration stimulus has good within-day repeatability in healthy volunteers, supporting its application in clinical studies and trials. We addressed the long-standing question of whether ROI- or voxel-based analysis is more accurate, predicting more robust estimation with the ROI-based approach, though underestimating CVR estimates with respect to the mean of the ground-truth distribution. Voxel-based analysis should be applied and interpreted with caution. Finally, we found that accurately modelling the CVR delay is key for reducing errors in CVR magnitude estimates.

## Data Availability

The datasets presented in this study can be found in online repositories. The names of the repository/repositories and accession number(s) can be found below: 3T BOLD CVR data from healthy volunteers suitable for within-day repeatability analyses (https://doi.org/10.7488/ds/3492).
