# Supplementary material for: Cerebrovascular reactivity measurements using 3T BOLD MRI and a fixed inhaled CO2 gas challenge: Repeatability and impact of processing strategy
Source: Front Physiol. 2023 Feb 6;14:1070233. doi: 10.3389/fphys.2023.1070233 (PMC9939770; doi:10.3389/fphys.2023.1070233)
Supplement: Supplementary file 1 [file DataSheet1.docx]

Supplementary Material

# CVR magnitudes and delays used for simulations

# The mean and standard deviation of CVR magnitudes and delays used in the simulations were extracted from the healthy volunteer data acquired for this study. For each participant, only one of the two scans was used and was chosen visually as the one with highest quality. The CVR and delay maps were then registered to the MNI template and averaged across participants. From the averaged maps, we extracted the distributions of CVR magnitudes and delays in NAWM (Figure S1), SGM and CGM using the MATLAB ‘fitdist’ function with a kernel distribution, excluding negative delays. We then generated ground-truth CVR magnitude and delay values for each simulated voxel by sampling the probability density functions; the true mean, median and standard deviation were calculated from the ground-truth data.

# Supplementary Figures


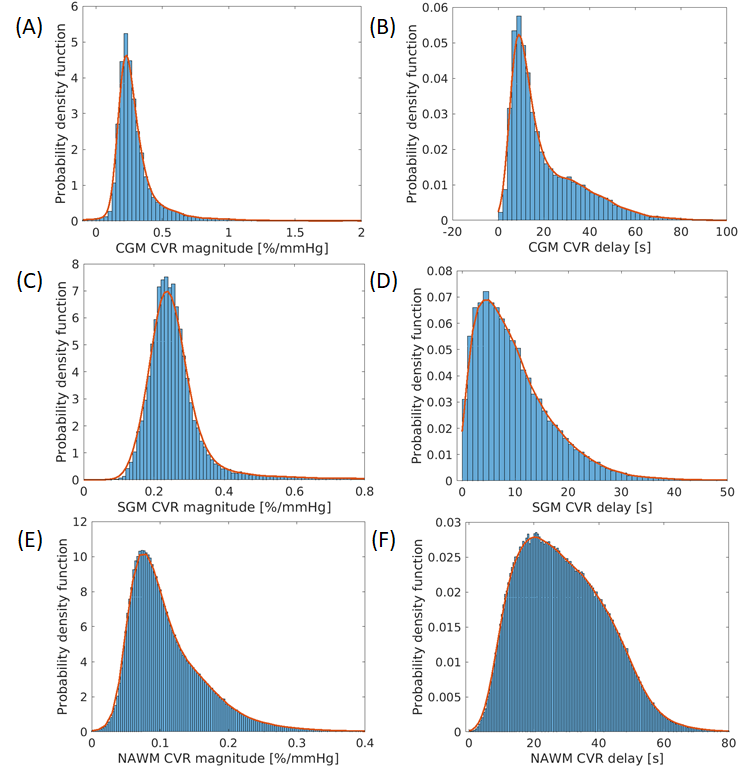


Figure S1. Distributions of CVR magnitudes (1^st^ column) and delays (2^nd^ column) in CGM (A, B), CGM (C, D) and NAWM (E, F) in the cohort-averaged parameter map. The orange curve represents the extracted distribution.

*
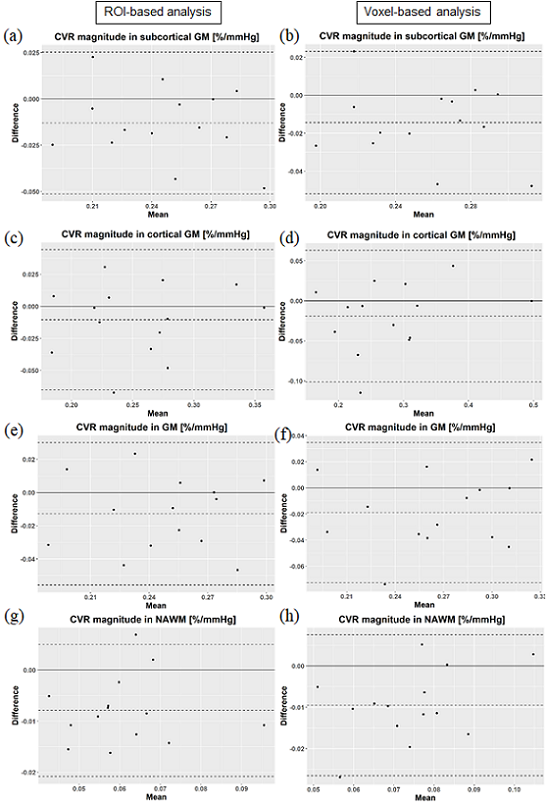
*

Figure S2. Comparison of CVR magnitudes between scans using Bland-Altman plots in subcortical GM (a, b), cortical GM (c, d), GM (e, f) and NAWM (g, h). CVR magnitudes were computed with the ROI-based (first column) and voxel-based analysis (second column). The dashed lines represent the mean inter-scan differences and the limits of agreement. (GM: grey matter, NAWM: normal-appearing white matter)


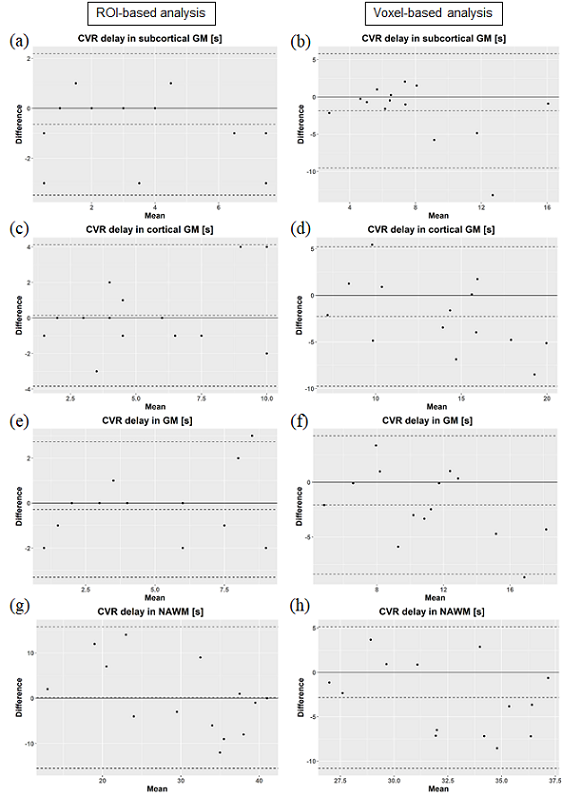


Figure S3. Comparison of CVR delays between scans using Bland-Altman statistics in subcortical GM (a, b), cortical GM (c, d), GM (e, f) and NAWM (g, h). CVR delays were computed with the ROI-based (first column) and voxel-based analysis (second column). The dashed lines represent the mean inter-scan differences and the limits of agreement. (GM: grey matter, NAWM: normal-appearing white matter)


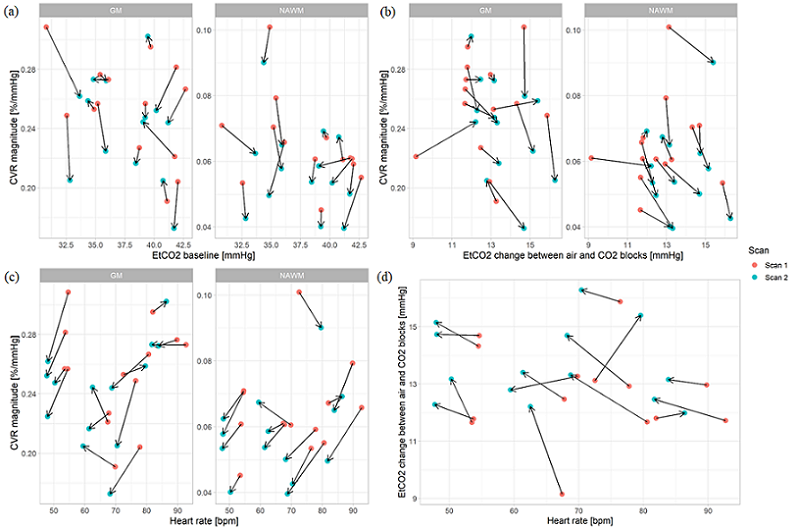


Figure S4. Difference in CVR magnitude and in physiological parameters between scans. Difference in CVR magnitude in GM and NAWM versus (a) the difference in EtCO2 baseline, (b) the difference in EtCO2 baseline, (c) the difference in heart rate. (d) Difference in EtCO2 change versus the difference in heart rate. (GM: grey matter, NAWM: normal-appearing white matter)

# Supplementary table

|  | ROI | Scan number | Delay constraint 1:  -31 to 93 s  (centre: 31 s) | | Delay constraint 2:  -31 to 124 s  (centre: 46.5 s) | | Inter-constraint difference  (constraint 2 − constraint 1) | |
| --- | --- | --- | --- | --- | --- | --- | --- | --- |
| Analysis type |  |  | Voxel | ROI | Voxel | ROI | Voxel | ROI |
| CVR magnitude [%/mmHg] | SGM | 1 | 0.26 ± 0.04 | 0.25 ± 0.04 | 0.26 ± 0.03 | 0.25 ± 0.04 | -0.008 ± 0.015  [-0.017, 0.001] | -0.006 ± 0.023  [-0.019, 0.007] |
|  |  | 2 | 0.25 ± 0.03 | 0.24 ± 0.03 | 0.25 ± 0.03 | 0.24 ± 0.03 | -0.003 ± 0.003  [-0.005, -0.002] | - |
|  | CGM | 1 | 0.29 ± 0.08 | 0.26 ± 0.05 | 0.27 ± 0.09 | 0.25 ± 0.06 | -0.020 ± 0.026  [-0.034, -0.005] | -0.007 ± 0.026  [-0.022, 0.008] |
|  |  | 2 | 0.27 ± 0.09 | 0.25 ± 0.05 | 0.26 ± 0.10 | 0.25 ± 0.05 | -0.011 ± 0.013  [-0.018, -0.004] | - |
|  | GM | 1 | 0.27 ± 0.04 | 0.25 ± 0.03 | 0.26 ± 0.05 | 0.25 ± 0.04 | -0.015 ± 0.021  [-0.027, -0.003] | -0.007 ± 0.025  [-0.021, 0.008] |
|  |  | 2 | 0.26 ± 0.05 | 0.24 ± 0.03 | 0.25 ± 0.05 | 0.24 ± 0.03 | -0.007 ± 0.008  [-0.012, -0.003] | - |
|  | NAWM | 1 | 0.08 ± 0.01 | 0.07 ± 0.01 | 0.08 ± 0.01 | 0.06 ± 0.01 | -0.001 ± 0.013  [-0.009, 0.006] | -0.002 ± 0.008  [-0.006, 0.002] |
|  |  | 2 | 0.07 ± 0.02 | 0.06 ± 0.01 | 0.07 ± 0.01 | 0.06 ± 0.01 | 0.002 ± 0.003  [-0.000, 0.004] | - |
| CVR delay [s] | SGM | 1 | 8.8 ± 4.8 | 3.9 ± 2.6 | 11.8 ± 6.9 | 3.9 ± 2.6 | 3.0 ± 2.6  [1.5, 4.5] | - |
|  |  | 2 | 6.9 ± 3.2 | 3.2 ± 2.4 | 9.2 ± 4.0 | 3.2 ± 2.4 | 2.3 ± 1.2  [1.6, 3.1] | - |
|  | CGM | 1 | 14.9 ± 5.3 | 5.4 ± 2.6 | 19.8 ± 6.7 | 5.4 ± 2.6 | 4.9 ± 2.4  [3.5, 6.3] | - |
|  |  | 2 | 12.7 ± 3.4 | 5.5 ± 3.3 | 18.2 ± 6.0 | 5.5 ± 3.3 | 5.6 ± 3.3  [3.6, 7.5] | - |
|  | GM | 1 | 12.2 ± 4.8 | 4.8 ± 2.7 | 16.4 ± 6.1 | 4.8 ± 2.7 | 4.2 ± 1.9  [3.1, 5.2] | - |
|  |  | 2 | 10.1 ± 3.3 | 4.5 ± 3.2 | 14.2 ± 5.1 | 4.5 ± 3.2 | 4.1 ± 2.3  [2.7, 5.4] | - |
|  | NAWM | 1 | 34.0 ± 4.6 | 30.1 ± 11.4 | 41.7 ± 5.1 | 29.5 ± 12.5 | 7.7 ± 1.9  [6.6, 8.8] | -0.6 ± 2.1  [-1.8, 0.7] |
|  |  | 2 | 31.2 ± 3.2 | 30.2 ± 7.4 | 39.3 ± 3.9 | 30.2 ± 7.4 | 8.1 ± 1.5  [7.2, 9.0] | - |
| Abbreviations - CVR: cerebrovascular reactivity, GM: grey matter, SGM: subcortical GM, CGM: cortical GM, NAWM: normal-appearing white matter, ROI: region of interest. | | | | | | | | |

Table S1. Mean and standard deviation across subjects of CVR magnitudes and delays in SGM, CGM, GM and NAWM computed for each delay constraint and each scan with ROI- and voxel-based processing. Mean and standard deviation of the inter-constraint differences are reported, with 95% confidence intervals for the mean given in square brackets.
